# Photobiomodulation Therapy Combined with Static Magnetic Field Reduces Pain in Patients with Chronic Nonspecific Neck and/or Shoulder Pain: A Randomized, Triple-Blinded, Placebo-Controlled Trial

**DOI:** 10.3390/life12050656

**Published:** 2022-04-29

**Authors:** Adeilson Matias Teixeira, Ernesto Cesar Pinto Leal-Junior, Heliodora Leão Casalechi, Adriane Aver Vanin, Paulo Roberto Vicente de Paiva, Fernando Hess Câmara Melo, Douglas Scott Johnson, Shaiane Silva Tomazoni

**Affiliations:** 1 Laboratory of Phototherapy and Innovative Technologies in Health (LaPIT), Post-Graduate Program in Rehabilitation Sciences, Nove de Julho University, São Paulo 01525-000, Brazil; adeilson.matias@uni9.edu.br (A.M.T.); ernesto.leal.junior@uninove.br (E.C.P.L.-J.); doraleao@gmail.com (H.L.C.); adrianevanin@yahoo.com.br (A.A.V.); pauloeducafisio@gmail.com (P.R.V.d.P.); hess.fernando@uni9.pro.br (F.H.C.M.); 2 Physiotherapy Research Group, Department of Global Public Health and Primary Care, University of Bergen, 5020 Bergen, Norway; 3 ELJ Consultancy, Scientific Consultants, Taubaté 12062-130, Brazil; 4 Multi Radiance Medical, Solon, OH 44139, USA; djohnson@multiradiance.com

**Keywords:** low-level laser therapy, light-emitting diode, neck pain, shoulder pain

## Abstract

Photobiomodulation therapy (PBMT) has been used to treat patients with chronic neck and/or shoulder pain. However, it is unknown whether the concurrent use of PBMT and static magnetic field (PBMT-sMF) also has positive effects in these patients. The aim of this study was to investigate the effects of PBMT-sMF versus placebo on pain intensity, range of motion (ROM) and treatment satisfaction in patients with chronic nonspecific neck and/or shoulder pain. A randomized controlled trial, with blinded assessors, therapists and patients was carried out. Seventy-two patients with chronic nonspecific neck and/or shoulder pain were randomized to either active PBMT-sMF (*n* = 36) or placebo PBMT-sMF (*n* = 36). Patients were treated twice weekly, over 3 weeks. Primary outcome was pain intensity, measured 15 min after the last treatment session and at 24-, 48-, 72-h, and 7-days after the last treatment. Secondary outcomes were ROM, patient’ treatment satisfaction, and adverse effects. PBMT-sMF was able to reduce pain intensity in all time points tested compared to placebo (*p* < 0.05). There was no difference between groups in the secondary outcomes (*p* > 0.05). Our results suggest that PBMT-sMF is better than placebo to reduce pain in patients with chronic nonspecific neck and/or shoulder pain at short-term.

## 1. Introduction

Pain in the neck and/or shoulder is one of the most common and prevalent condition worldwide [1,2,3]. Patients with neck and/or shoulder pain usually can present sleep disturbance and sitting tolerance. In addition, they can have reduced neck and shoulder range of motion and poor quality of life. All of these aspects contribute to a high work absenteeism [4]. According to the Global Burden of the Disease, it is estimated that neck and/or shoulder pain is the fourth condition in terms of years lived with disability worldwide [5], generating high healthcare costs [6]. Neck and/or shoulder pain can be related to an identifiable musculoskeletal pathology (specific), compression, or injury of the peripheral nervous system (neuropathic) or an unidentified cause (nonspecific) [3,7,8]. The prognosis of neck and/or shoulder pain usually is good, however less favorable in patients with chronic pain (>12 weeks) [9], requiring use of pain management strategies. Patients with chronic neck and/or shoulder pain can benefit from multimodal approach, including education and advice, mobilization and manipulation, exercise program, transcutaneous electrical nerve stimulation, dry needling, traction, and photobiomodulation therapy (PBMT) [10].

PBMT is a non-thermal light therapy that consists of the application of non-ionizing forms of lights sources such as light amplification by the stimulated emission of radiation (laser) and light-emitting diode (LED) to promote photophysical and photochemical events on biological tissues [11]. These photochemical and photobiological effects of PBMT are related to the enhancement of energy production, decrease of oxidative stress and prevention of cell death [12,13]. PBMT can be used alone or combined with another therapeutic agent such static magnetic field (sMF), for instance. The concurrent use of these therapeutic agents aims to increase the positive effects triggered, through the synergy between them [14,15]. PBMT combined with static magnetic field (PBMT-sMF) has been used in the same device to treat pain in different musculoskeletal conditions such as knee pain, postoperative pain after total hip arthroplasty, temporomandibular disorders, low back pain, osteoarthritis, and also fibromyalgia [16,17,18,19,20,21]. It has been demonstrated that PBMT-sMF applied with optimized parameters and doses within a therapeutic window has positive effects in managing pain of musculoskeletal conditions.

There is evidence about the effects of PBMT alone to treat patients with chronic neck and shoulder pain. A systematic review and meta-analysis showed that pain intensity was reduced in these patients after treatment with PBMT alone [22]. This reduction remained up to 22 weeks after the end of the treatment. In addition, another more recent systematic review found moderate quality of evidence in favor of PBMT for chronic neck pain, demonstrating that new clinical trials would be important and beneficial for the field [23].

Although there is evidence about the effects of PBMT alone on chronic neck and/or shoulder pain, to date, there are no studies demonstrating the effects of PBMT-sMF on pain intensity of patients with chronic neck and/or shoulder pain. It is unknown whether the concurrent use of PBMT and sMF is able to reduce pain intensity in these patients. Furthermore, it is unknown whether PBMT-sMF can increase the effect size on pain intensity in patients with neck and/or shoulder pain due the synergistic effect between these therapeutic agents. Therefore, the investigation about the effects of PBMT-sMF could be able to establish this therapeutic approach as an alternative to treat patients with chronic nonspecific neck and/or shoulder pain.

We hypothesized that PBMT-sMF would have advantage over placebo to reduce pain intensity and increase range of motion (ROM) in patients with chronic nonspecific neck and/or shoulder pain. Therefore, this study aimed to investigate the effects of PBMT-sMF versus placebo on pain intensity, ROM and treatment satisfaction in patients with chronic nonspecific neck and/or shoulder pain.

## 2. Materials and Methods

### 2.1. Study Design and Ethical Aspects

A registered (NCT02940119), parallel randomized, triple-blinded (assessors, therapists and patients), placebo-controlled trial was performed at Laboratory of Phototherapy and Innovative Technologies in Health (LaPIT), São Paulo, Brazil. This study was approved by the Research Ethics Committee of Nove de Julho University (#1409527). All eligible patients signed written informed consent prior to the study start.

### 2.2. Eligibility Criteria

The inclusion criteria were: patients with chronic nonspecific neck and/or shoulder pain, defined as pain in the neck and shoulder regions, persisting over at least 3 months and without a specific etiology [24]; of any gender; aged of 18 years or older; with pain intensity of at least 50 mm (measured by 0–100 mm visual analog scale); and fluent in Portuguese (reading and writing).

The exclusion criteria were: patients presenting nerve root compromise, cervical spinal canal stenosis, and cervical spondylosis/cervical osteoarthritis; neck pain caused by diseases such as rheumatoid arthritis, meningitis or cancer/tumors; use of local corticosteroids/botulin toxin injection within the prior 30 days; fibromyalgia; diabetic neuropathic pain; surgery to the neck and/or shoulder region in the past 12 months; pregnant; and breast feeding.

### 2.3. Randomization and Blinding Procedures

Randomization was generated through the random.org website and performed by a researcher not involved with any phase of the study. It was generated a simple randomization with an allocation ratio of 1:1. The same researcher programmed the device as active PBMT-sMF or placebo PBMT-sMF and coding the treatments according to the prior randomization. This researcher was instructed not to disclose the programmed intervention and codes to the assessors, therapists or any of the patients and other researchers involved in the study until its completion. Concealed allocation was achieved through the use of sequentially numbered, sealed, and opaque envelopes. Prior to initiation of treatment, patients were allocated to their respective intervention groups (active PBMT-sMF or placebo PBMT-sMF).

The assessors, therapists, and patients were blinded throughout the study. To ensure complete blinding, the device used had no thermal effects [25], and emitted the same sounds, lights, and information on the display regardless of the mode used.

### 2.4. Interventions

**Figure 1:**
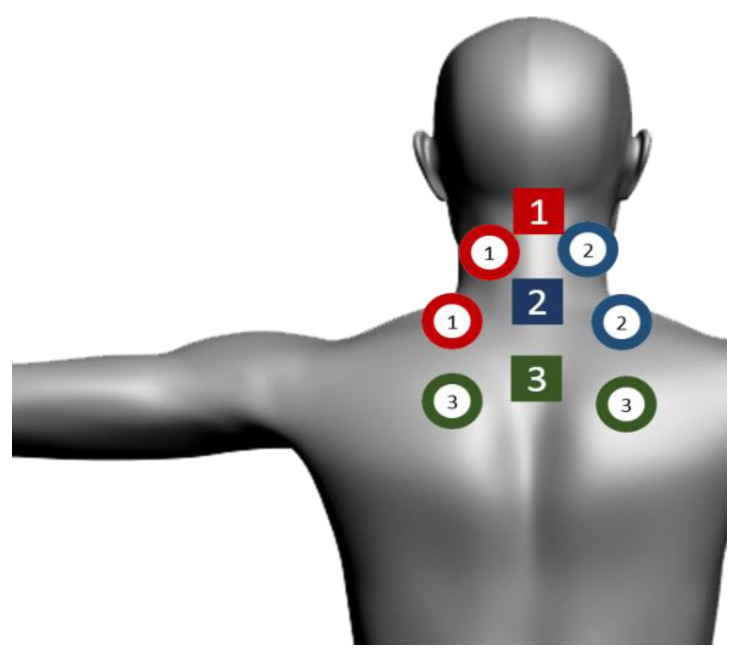
Sites of PBMT-sMF irradiation. The neck and shoulder region were separated in three zones for the irradiation of PBMT-sMF. The SE25 plus LS50 emitters were positioned over the first target area: spinous processes of C3, left elevator scapulae, left trapezius (1). The SE25 plus LS50 emitters were then positioned over the second target area: spinous processes of C7, right elevator scapulae, right trapezius (2). The SE25 plus LS50 emitters were then positioned over the third and final target area: spinous process of T4, left rhomboid major, and right rhomboid major (3). Circles represent the LS50 emitter and squares represent the SE25 emitters.

The active and placebo PBMT-sMF were performed using the same device and the irradiated sites were the same for both therapies: 9 sites on the neck and shoulder region. The neck and shoulder region were separated in three zones (Zone 1, Zone 2, and Zone 3) for the irradiation of PBMT-sMF or placebo PBMT-sMF, in order to sufficiently cover the whole painful area. The SE25 plus LS50 emitters were positioned over the first target area (Zone 1): spinous processes of C3, left elevator scapulae, left trapezius. The SE25 plus LS50 emitters were then positioned over the second target area (Zone 2): spinous processes of C7, right elevator scapulae, right trapezius. The SE25 plus LS50 emitters were then positioned over the third and final target area (Zone 3): spinous process of T4, left rhomboid major, and right rhomboid major (Figure 1). The total procedure administration duration was 9 min (3 min per zone). The emitters were not moved during the procedure administration. Patients underwent the treatment twice a week at approximately the same time of the day, with intervals of 3 to 4 days between sessions, over 3 weeks, yielding 6 treatments.

Intervention specifications included:

(1) Active PBMT-sMF group: Active PBMT-sMF was delivered by the MR4^®^ Laser Therapy Systems manufactured by Multi Radiance Medical, Solon, OH, USA, using SE25 and LaserShower (LS50) as emitters. The full description of parameters is provided in Table 1. At each treatment session, patients received a total dose of 230.85 J. At the end of the 6 treatment sessions, patients received a total dose of 1385.10 J. The parameters were based on clinical treatments performed by members of the manufacturer’s medical advisory board and following the manufacturer’s recommendation. The optical power of the device was checked by a Thorlabs thermal power meter (Model S322C, Thorlabs^®^, Newton, NJ, USA) before the beginning of the study and after the study was completed.

(2) Placebo PBMT-sMF group: The placebo treatment was delivered using the same device that active PBMT-sMF but without any emission of therapeutic dose. In the placebo mode, the only visible diode was the red one. For the placebo mode, the 905 nm laser diodes and the 875 nm LED diodes were turned off. In addition, the power of the 640 nm LED diodes was turned off to 1 mW (for each diode) to keep the visual aspect of the red light. Finally, in the placebo mode, the sMF was also turned off.

### 2.5. Outcomes

The primary outcome was pain intensity measured by a visual analog scale (VAS)-100 mm scale. Patients were instructed to report the pain intensity perceived in the neck and shoulder region based on a 0–100 mm-scale, with 0 (zero) being “no pain” and 100 being “the worst possible pain”.

The secondary outcomes were active range of motion (ROM) (in all motions) of cervical and shoulder, measured by a blinded assessor using goniometer. In addition, patient’ satisfaction with the treatment, measured by a Likert scale ranging from “very satisfied”, “somewhat satisfied”, “neither satisfied nor dissatisfied”, “not very satisfied” to “not at all satisfied”. Finally, adverse effects were collected by a report.

### 2.6. Procedures

**Figure 2:**
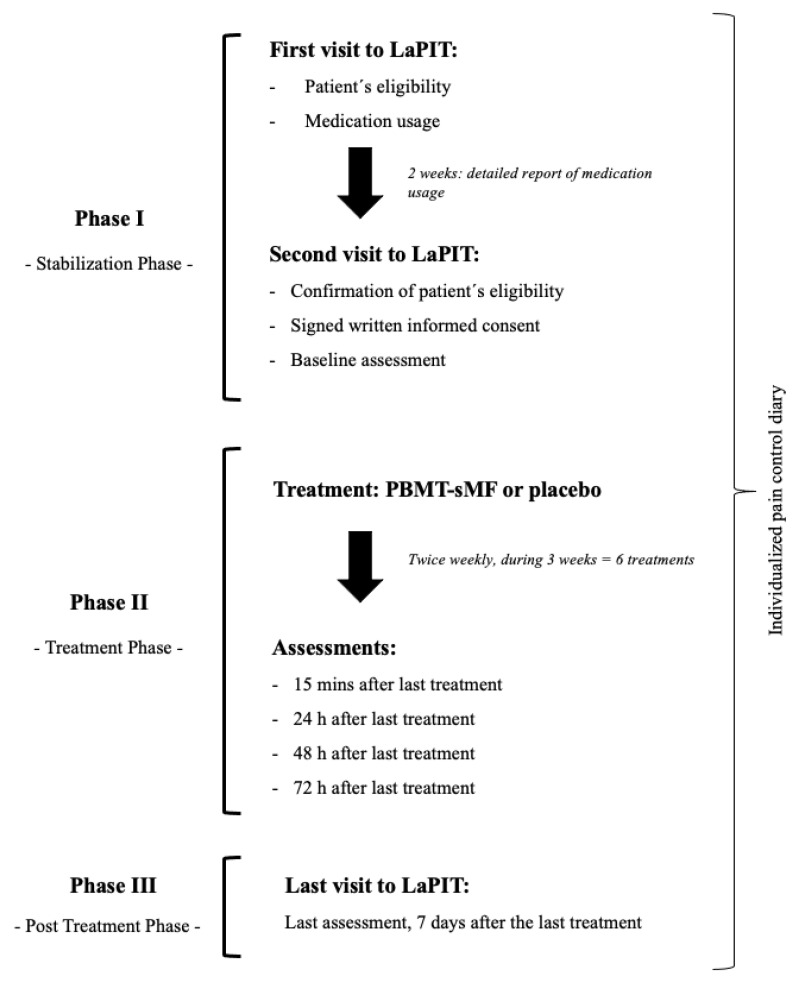
Procedures of the study.

The study was divided into three separate phases and the outcomes were collected during each of these phases (Figure 2). The phase 1 was the Stabilization Phase which determined the patients’ eligibility and a detailed report of medication usage (over 2 weeks). In addition, at baseline assessment were collected the pain intensity, measured by VAS-100 mm and active ROM, measured by a goniometer. The phase 2 was the Treatment Phase which patients underwent the treatment (active PBMT-sMF or placebo PBMT-sMF). In this phase, the assessment of pain intensity was collected at fifteen minutes after the last treatment session and at 24-, 48-, and 72-h after the last treatment session. In addition, active ROM and patient’s satisfaction with the treatment were collected 15 min after the last treatment session and the medication usage was recorded. The phase 3 was the Post Treatment Phase in which patients returned to the LaPIT for a final follow up 7 days after the last treatment. In this phase, the assessment of pain intensity, active ROM, patient’ satisfaction, and medication usage were collected as well. Patients were allowed to maintain pain control throughout the study with the use of medications and/or concomitant treatments (used in phase 1) of the study. However, it was requested to record it in an individualized pain control diary.

### 2.7. Sample Size

The success criterion was predefined as a 30% or greater reduction in the pain intensity from baseline to the final evaluation. The overall efficacy of the PBMT-sMF therapy was considered successful if a minimal difference of 35% is found between the active and placebo group. It was anticipated that about 55% of patients in the active group and about 20% of patients in the placebo group would meet the individual success criteria and intended application of a two-tailed test with an alpha of 0.05 and power of 0.8. A sample size of 33 eligible participants per group was calculated [26] and a patient loss-to-follow-up of 10% was anticipated to bring the sample size to 36 patients in each group. Therefore, a total of 72 patients were enrolled in this study and stratified by Fitzpatrick skin type as following: Lighter-skinned individuals were grouped into Fitzpatrick skin types I, II, and III, while darker-skinned individuals were grouped into Fitzpatrick skin types IV, V, and VI.

### 2.8. Statistical Analysis

The statistical analysis was conducted following intention-to-treat principles [27]. The Fisher’s Exact Test for two independent groups was conducted to compare the proportion of successes between procedure groups. To analyze intensity of pain, a one-way ANCOVA for each of the PBMT-sMF and placebo groups was performed with the sub-analysis of the subject’s stratification to Fitzpatrick skin types. For secondary outcomes, a series of *t*-tests for independent samples were performed and the level of statistical significance was set at *p* < 0.05. The data were presented in absolute values and percentage of change.

## 3. Results

**Figure 3:**
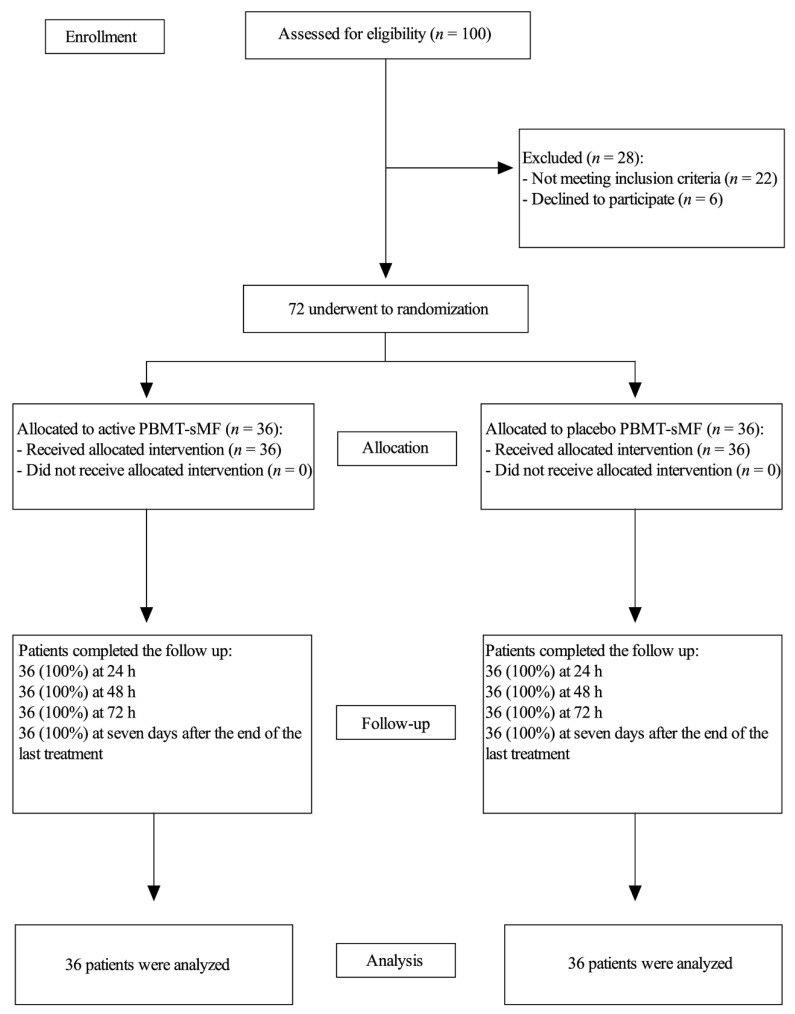
Enrollment and randomization.

In total, 72 patients (proportion of female to male in the study groups was 24:12 for the active PBMT-sMF and 23:13 for the placebo) were recruited and completed all procedures between November 2016 and Abril 2017 (Figure 3). Proportion of lighter to darker skinned individuals according to Fitzpatrick skin types was 22:14 for both active PBMT-sMF and placebo PBMT-sMF groups per sample stratification. The baseline characteristics of both groups were similar (*p* > 0.05) (Table 2).

In active PBMT-sMF group 100% (*n* = 36) of patients met the study individual subject success criteria, i.e., 36 patients had 30% or greater reduction in the pain intensity from baseline to the final evaluation. In the placebo PBMT-sMF group 61% (*n* = 22) of patients met the study individual subject success criteria.

Patients allocated to the PBMT-sMF group had a greater magnitude of the change on pain intensity in neck and shoulder when compared to the placebo PBMT-sMF group (*p* < 0.0001) at 15 min post-intervention (Table 3).

In addition, patients allocated to the PBMT-sMF group had reduced pain intensity compared to the placebo PBMT-sMF group (*p* < 0.05) at 24-, 48-, 72-h, and 7 days post-intervention (Table 4).

**Figure 4:**
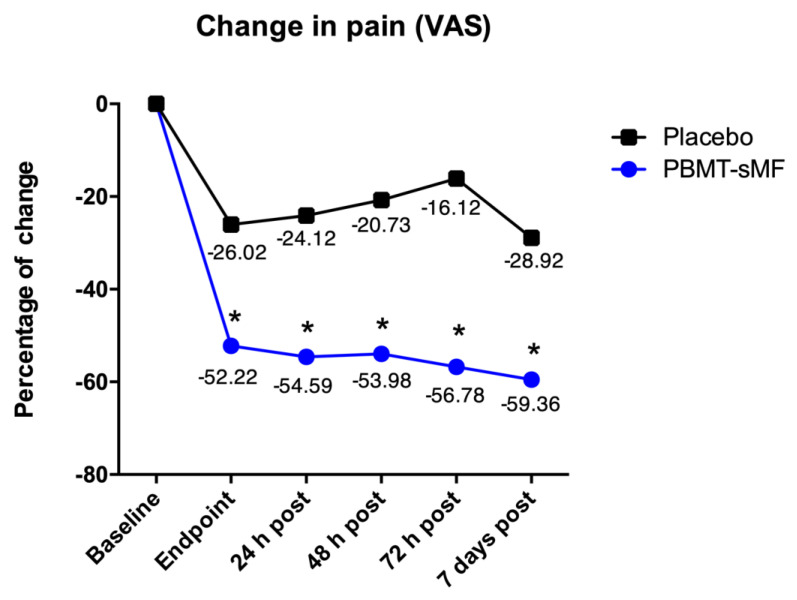
Change on intensity of pain measured by VAS. Patients allocated to the PBMT-sMF group decreased intensity of pain compared to patients allocated to the placebo PBMT-sMF group (* *p* < 0.05).

The change on intensity of pain measured by VAS demonstrated that patients allocated to the PBMT-sMF group reduced intensity of pain compared to patients allocated to the placebo PBMT-sMF group in all time points (*p* < 0.05) (Figure 4).

ROM measurements increased for both groups, however there was no difference between PBMT-sMF and placebo PBMT-sMF groups (*p* > 0.05) in all time points tested (Table 5).

There was no difference between PBMT-sMF and placebo PBMT-sMF groups regarding the outcome patients’ satisfaction with the treatment (*p* > 0.05). 83% (*n* = 30) of patients from the PBMT-sMF group and 83% (*n* = 30) of patients of the placebo PBMT-sMF group reported being “Satisfied” (Very Satisfied or Somewhat Satisfied) at the end of the treatment. In addition, 86% (*n* = 31) of patients from PBMT-sMF group and 89% (*n* = 32) of patients of the placebo PBMT-sMF group reported being “Satisfied” (Very Satisfied or Somewhat Satisfied) in the last follow up assessment.

There was no difference between PBMT-sMF and placebo PBMT-sMF groups regarding medication usage (*p* > 0.05). In total, 11% (*n* = 4) of patients from the PBMT-sMF and 3% (*n* = 1) of patients from placebo PBMT-sMF group did not use any medication during the Stabilization Phase (phase 1). In total, 22% (*n* = 8) of patients from the PBMT-sMF group and 17% (*n* = 6) of patients from placebo PBMT-sMF group did not use any medication during the Treatment Phase (phase 2). Finally, 53% (*n* = 19) of patients from PBMT-sMF and 31% (*n* = 11) of patients from the placebo PBMT-sMF group did not use any medication during the Post Treatment Phase (phase 3).

Patients did not report any adverse effects.

## 4. Discussion

This is the first triple-blinded randomized controlled trial that investigated the effects of the concurrent use of PBMT and sMF (PBMT-sMF) compared to placebo in patients with chronic nonspecific neck and/or shoulder pain. PBMT-sMF was able to decrease pain intensity in all time points tested compared to placebo. Moreover, we observed that ROM measurements increased in both groups, however there was no difference between PBMT-sMF and placebo groups. Finally, there was no difference between groups on patients’ satisfaction with the treatment and medication usage.

It was previously demonstrated that PBMT alone (904 nm wavelength, dose of 2 J/cm^2^, 3 min of irradiation over the trigger points, in 10 sessions of treatment) was able to reduce pain intensity at rest and at movement after the end of the treatment and 10 weeks after the end of the treatment [28]. In addition, another clinical trial demonstrated that PBMT (780 nm wavelength, 5 J/cm^2^, 3 min and 16 s of irradiation) associated with stretching was superior to stretching alone in reducing pain intensity after 10 sessions of treatment and 3 weeks after the end of the treatment [29]. Despite the difference about the parameters of PBMT used in the abovementioned studies and ours, we observed a reduction on pain intensity as well. Therefore, our findings suggest that the effects of PBMT-sMF, applied with multi-wavelength, are similar to the PBMT alone to treat patients with chronic neck and/or shoulder pain, even with less sessions of treatment (6 sessions in total).

In contrast, a clinical trial demonstrated that PBMT alone (830 nm wavelength, energy of 7 J per site, 6 sites of irradiation, and 2 min of irradiation over each site in 15 sessions of treatment) was no different to placebo in the treatment of patients with chronic neck pain. Although both groups had an improvement in pain intensity, ROM and functional disability, PBMT was not superior to placebo [30]. These findings are contrary to ours with regard to pain intensity. However, our results are in line with the best available evidence, through a systematic review with meta-analysis [22]. On the other hand, we observed that PBMT-sMF was not able to improve ROM, as well as the abovementioned study, suggesting that neither PBMT or PBMT-sMF have positive effects on ROM of patients with chronic neck and/or shoulder pain.

We observed that PBMT-sMF was able to reduce pain in patients with chronic neck and/or shoulder pain when compared to placebo, however it was not enough to have a positive impact on ROM. We believe that in order to increase ROM, PBMT-sMF would need to increase the extensibility of the different tissues in the neck and shoulder area, which is not supported by the current evidence. These aspects can partially explain the results observed in our study. Thus, the results found in this clinical trial support the use of PBMT-sMF only to reduce pain intensity, but not to improve ROM in patients with chronic neck and/or shoulder pain.

One of the strengths of this clinical trial was that is had a low risk of bias, since we used true randomization, concealed allocation, intention-to-treat analysis, and assessors, therapists, and patients were blinded. In addition, we used a placebo group to control possible confounding factors. Moreover, we controlled for cointerventions in both groups through an individualized patient diary used during the whole trial. Finally, we had no drop-out or loss to follow-up. On the other hand, we can consider a limitation of this clinical trial was not having measured disability through a disability scale. In addition, we only measured the short-term effects of PBMT-sMF.

Further randomized controlled trials are needed to investigate the medium- and long-term effects of PBMT-sMF in patients with chronic nonspecific neck and/or shoulder pain. In addition, it is important to investigate the effects of PBMT-sMF also on the disability of these patients.

## 5. Conclusions

There is an advantage in the use of PBMT-sMF to reduce pain intensity in patients with chronic nonspecific neck and/or shoulder pain in the short-term. However, there is no advantage in the use of PBMT-sMF to increase ROM and patient treatment satisfaction.

## Figures and Tables

**Table 1:** Parameters for SE25™ and LaserShower™ cluster probes.

	SE25	LaserShower
Number of lasers	1 Super-pulsed infrared	4 Super-pulsed infrared
Wavelength (nm)	905 (±1)	905 (±1)
Frequency (Hz)	3000	3000
Peak power (W)-each	25	12.50
Average mean optical output (mW)-each	7.5	3.75
Power density (mW/cm^2^)-each	17.05	8.52
Energy density (J/cm^2^)-each	3.07	1.53
Dose (J)-each	1.35	0.675
Spot size of laser (cm^2^)-each	0.44	0.44
Number of red LEDs	4 Red	4 Red
Wavelength of red LEDs (nm)	640 (±10)	640 (±10)
Frequency (Hz)	2	2
Average optical output (mW)-each	15	15
Power density (mW/cm^2^)-each	16.67	16.67
Energy density (J/cm^2^)-each	3	3
Dose (J)-each	2.7	2.7
Spot size of red LED (cm^2^)-each	0.9	0.9
Number of infrared LEDs	4 Infrared	4 Infrared
Wavelength of infrared LEDs (nm)	875 (±10)	875 (±10)
Frequency (Hz)	16	16
Average optical output (mW)-each	17.5	17.5
Power density (mW/cm^2^)-each	19.44	19.44
Energy density (J/cm^2^)-each	3.5	3.5
Dose (J)-each	3.15	3.15
Spot Size of LED (cm^2^)-each	0.9	0.9
Number of magnets	1	1
Magnetic Field (mT)	35	35
Irradiation time per site (sec)	180	180
Total dose per site (J)	24.75	26.10
Aperture of device (cm^2^)	4	20
Total dose applied per treatment session (J)	230.85 J	
Application mode	Cluster probe held stationary in skin contact with a 90-degree angle and slight pressure	Cluster probe held stationary in skin contact with a 90-degree angle and slight pressure

**Table 2:** Demographic and clinical characteristics of the patients at baseline (*n* = 72).

Variables	Active PBMT-sMF (*n* = 36)	Placebo PBMT-sMF (*n* = 36)
Age (y)	32.78 (9.99)	31.39 (9.90)
Gender (%)		
Female	24 (66.7)	23 (63.9)
Male	12 (33.3)	13 (36.1)
Fitzpatrick skin type (%)		
Lighter skinned	22 (61.1)	22 (61.1)
Darker skinned	14 (38.9)	14 (38.9)
Weight (kg)	71.92 (14.19)	76.35 (16.02)
Height (cm)	167.6 (8.59)	167.1 (10.35)
Individual subject success criteria (%)	36 (100)	22 (61)
Pain intensity (0–100)	68.42 (11.31)	70.31 (13.44)
ROM (°)		
Neck		
Flexion	54.03 (15.20)	52.81 (10.78)
Extension	46.67 (12.54)	51.03 (11.10)
Lateral (right)	40.69 (11.45)	39.50 (9.72)
Lateral (left)	41.89 (11.29)	38.00 (7.86)
Rotation (right)	57.64 (11.05)	63.42 (11.79)
Rotation (left)	63.28 (8.92)	67.36 (9.75)
Shoulder		
Flexion (right)	159.58 (17.97)	164.25 (19.23)
Flexion (left)	160.17 (19.52)	166.58 (14.27)
Extension (right)	50.08 (9.45)	48.75 (11.67)
Extension (left)	52.08 (12.78)	50.36 (13.84)
External rotation (right)	76.11 (11.66)	73.69 (10.86)
External rotation (left)	71.81 (13.07)	74.36 (12.35)
Internal rotation (right)	76.81 (12.91)	76.64 (11.79)
Internal rotation (left)	78.25 (8.91)	77.92 (11.55)
Abduction (right)	147.47 (26.48)	156.86 (26.48)
Abduction (left)	149.39 (22.62)	162.44 (20.88)

Categorical variables are expressed as number (%). Continuous variables are expressed as mean (SD). PBMT-sMF, photobiomodulation therapy combined with static magnetic field.

**Table 3:** Magnitude of the change on pain intensity in neck and shoulder.

Pain Intensity (VAS)	Active PBMT-sMF (*n* = 36)	Placebo PBMT-sMF (*n* = 36)
Baseline	68.42 (64.59 to −72.24)	70.31 (65.76 to −74.85)
15 min post-intervention	16.19 (12.59 to −19.78)	44.29 (39.03 to −49.54)
Change	−52.23 (−56.79 to −47.60)	−26.02 (−31.50 to −20.53)

Data are expressed in mean and 95% confidence intervals (CI).

**Table 4:** Pain intensity measured by VAS.

Time Points	Active PBMT/sMF (*n* = 36)	Placebo PBMT-sMF (*n* = 36)
Baseline	68.42 (64.59 to −72.24)	70.31 (65.76 to −74.85)
15 min post-intervention	16.19 (12.59 to −19.78)	44.29 (39.03 to −49.54)
24 h post-intervention	13.83 (9.67 to −17.98)	46.19 (42.26 to −50.11)
48 h post-intervention	14.44 (9.63 to −19.24)	49.58 (44.52 to −54.63)
72 h post-intervention	11.64 (7.18 to −16.09)	54.19 (46.98 to −61.39)
7 days post-intervention	9.06 (5.47 to −12.64)	41.39 (34.67 to −48.10)

Data are expressed in mean and 95% confidence intervals (CI).

**Table 5:** Neck and shoulder ROM across evaluation points.

ROM (°)		Active PBMT-sMF (*n* = 36)	Placebo PBMT-sMF (*n* = 36)
***Neck***			
Flexion	Baseline 15 min post-intervention 7 days post-intervention	54.03 (48.88–59.17) 55.81 (51.59–60.02) 59.33 (56.19–62.46)	52.81 (49.16–56.45) 57.33 (54.05–60.60) 56.81 (53.33–60.28)
Extension	Baseline 15 min post-intervention 7 days post-intervention	46.67 (42.42–50–91) 54.17 (50.40–57.93) 55.56 (52.40–58.71)	51.03 (47.27–54.78) 56.14 (53.15–59.12) 57.64 (54.48–60.79)
Lateral—Right	Baseline 15 min post-intervention 7 days post-intervention	40.69 (36.81–44.56) 45.19 (42.21–48.16) 44.33 (41.07–47.58)	39.50 (36.21–42.78) 42.36 (39.38–45.33) 42.03 (38.87–45.18)
Lateral—Left	Baseline 15 min post-intervention 7 days post-Intervention	41.89 (38.07–45.70) 42.36 (39.90–44.81) 43.17 (41.09–45.24)	38.00 (35.34–40.65) 43.22 (40.46–45.97) 44.61 (41.54–47.67)
Rotation—Right	Baseline 15 min post-intervention 7 days post-intervention	57.64 (53.90–61.37) 70.97 (67.66–74.27) 69.97 (66.18–73.75)	63.42 (59.43–67.40) 68.56 (65.28–71.83) 67.86 (63.78–71.93)
Rotation—Left	Baseline 15 min post-intervention 7 days post-intervention	63.28 (60.26–66.29) 74.67 (71.35–77.98) 69.67 (65.27–74.06)	67.36 (64.06–70.65) 71.47 (67.69–75.24) 71.11 (67.02–75.19)
***Shoulder***			
Flexion—Right	Baseline 15 min post-intervention 7 days post-intervention	159.58 (153.49–165.66) 166.44 (161.37–171.50) 170.83 (167.38–174.27)	164.25 (157.74–170.75) 169.31 (165.10–173.51) 171.53 (167.78–175.27)
Flexion—Left	Baseline 15 min post-intervention 7 days post-intervention	160.17 (152.56–167.77) 170.14 (164.89–175.38) 173.47 (169.23–177.70)	166.58 (161.75–171.40) 171.47 (167.71–175.22) 172.08 (168.81–175.34)
Extension—Right	Baseline 15 min post-intervention 7 days post-intervention	50.08 (46.88–53.27) 56.06 (51.87–60.24) 54.03 (50.22–57.83)	48.75 (44.80–52.69) 53.47 (49.63–57.30) 52.36 (48.09–56.62)
Extension—Left	Baseline 15 min post-intervention 7 days post-intervention	52.08 (47.75–56.40) 55.14 (51.37–58.90) 55.31 (51.87–58.74)	50.36 (45.67–55.04) 56.53 (52.65–60.40) 57.36 (53.67–61.04)
External Rotation—Right	Baseline 15 min post-intervention 7 days post-intervention	76.11 (72.16–80.05) 81.89 (79.10–84.67) 82.78 (79.22–86.33)	73.69 (70.01–77.36) 81.11 (77.42~84.79) 81.86 (77.14–86.67)
External Rotation—Left	Baseline 15 min post-intervention 7 days post-intervention	71.81 (67.38–76.23) 80.69 (76.84–84.53) 82.78 (78.91–86.64)	74.36 (70.12–78.59) 78.83 (74.56–83.09) 79.06 (74.67–83.44)
Internal Rotation—Right	Baseline 15 min post-intervention 7 days post-intervention	76.81 (72.44–81.17) 78.47 (74.17–82.76) 84.03 (80.55–87.50)	76.64 (72.65–80.62) 76.86 (72.43–81.28) 82.31 (78.88–85.73)
Internal Rotation—Left	Baseline 15 min post-intervention 7 days post-intervention	78.25 (75.23–81.26) 77.56 (73.58–81.53) 84.44 (90.98–87.89)	77.92 (74.01–81.82) 80.69 (76.74–84.63) 83.33 (79.66–86.99)
Abduction—Right	Baseline 15 min post-intervention 7 days post-intervention	147.47 (138.51–156.42) 161.67 (155.28–168.05) 165.83 (159.99–171.66)	156.86 (147.90–165.81) 161.28 (154.07–168.48) 162.44 (155.06–169.81)
Abduction—Left	Baseline 15 min post-intervention 7 days post-intervention	149.39 (141.73–157.01) 163.89 (156.83–170.94) 167.22 (160.92–173.51)	162.44 (155.40–169.47) 163.61 (156.20–171.91) 165.67 (157.70–173.63)

Data are expressed in mean and 95% confidence intervals (CI).

## Data Availability

The datasets generated and analyzed during the current study are available from the corresponding author on reasonable request.
